# Dr. Alonzo Churchill

**Published:** 1897-02

**Authors:** 


Dr. Alonzo Churchill, of Utica, died recently at the advanced age of eighty-six. Dr. Churchill had been in active practice for more than fifty years. He served as medical officer during the late civil war and was left at Savage's Station, Va., in 1862, to assist in caring for the wounded that accumulated during the seven days' battles round Richmond, when the Army of the Potomac was retiring to Harrison's Landing.

Dr. Churchill was a capable physician and a useful citizen, whose courtly manners distinguished him as a gentleman of the old school, a type now rapidly disappearing.

